# Recent trends in wearable device used to detect freezing of gait and falls in people with Parkinson’s disease: A systematic review

**DOI:** 10.3389/fnagi.2023.1119956

**Published:** 2023-02-15

**Authors:** Tinghuai Huang, Meng Li, Jianwei Huang

**Affiliations:** ^1^Laboratory of Laser Sports Medicine, South China Normal University, Guangzhou, Guangdong, China; ^2^Department of Gastroenterology, The Fifth Affiliated Hospital of Guangzhou Medical University, Guangzhou Medical University, Guangzhou, Guangdong, China

**Keywords:** wearable device, Parkinson’s disease, freezing of gait (FOG), fall – Wound, FOG detection algorithm

## Abstract

**Background:**

The occurrence of freezing of gait (FOG) is often observed in moderate to last-stage Parkinson’s disease (PD), leading to a high risk of falls. The emergence of the wearable device has offered the possibility of FOG detection and falls of patients with PD allowing high validation in a low-cost way.

**Objective:**

This systematic review seeks to provide a comprehensive overview of existing literature to establish the forefront of sensors type, placement and algorithm to detect FOG and falls among patients with PD.

**Methods:**

Two electronic databases were screened by title and abstract to summarize the state of art on FOG and fall detection with any wearable technology among patients with PD. To be eligible for inclusion, papers were required to be full-text articles published in English, and the last search was completed on September 26, 2022. Studies were excluded if they; (i) only examined cueing function for FOG, (ii) only used non-wearable devices to detect or predict FOG or falls, and (iii) did not provide sufficient details about the study design and results. A total of 1,748 articles were retrieved from two databases. However, only 75 articles were deemed to meet the inclusion criteria according to the title, abstract and full-text reviewed. Variable was extracted from chosen research, including authorship, details of the experimental object, type of sensor, device location, activities, year of publication, evaluation in real-time, the algorithm and detection performance.

**Results:**

A total of 72 on FOG detection and 3 on fall detection were selected for data extraction. There were wide varieties of the studied population (from 1 to 131), type of sensor, placement and algorithm. The thigh and ankle were the most popular device location, and the combination of accelerometer and gyroscope was the most frequently used inertial measurement unit (IMU). Furthermore, 41.3% of the studies used the dataset as a resource to examine the validity of their algorithm. The results also showed that increasingly complex machine-learning algorithms had become the trend in FOG and fall detection.

**Conclusion:**

These data support the application of the wearable device to access FOG and falls among patients with PD and controls. Machine learning algorithms and multiple types of sensors have become the recent trend in this field. Future work should consider an adequate sample size, and the experiment should be performed in a free-living environment. Moreover, a consensus on provoking FOG/fall, methods of assessing validity and algorithm are necessary.

**Systematic Review Registration:** PROSPERO, identifier CRD42022370911.

## Introduction

Parkinson’s disease (PD) is an age-related progressive neurodegenerative condition clinically characterized by bradykinesia and either resting tremor or rigidity, affecting about 1% of adults older than 60 worldwide (Samii et al., 2004). The freezing of gait (FOG) occurrence is often observed in moderate to last-stage PD, increasing fall risk, reducing the quality of life, and the likelihood of independent living (Kerr et al., 2010).

As a complex and highly-variable phenomenon, FOG can be defined as a brief episode absence or marked reduction in the forward progression of the feet despite the intention to walk, which remains a persistent and incapacitating motor problem for many patients in daily life (Rahman et al., 2008). Episodes can be brief or exceed 30 s (Schaafsma et al., 2003). It is hard to anticipate the occurrence of FOG for patients with PD who live at home since FOG can occur several times a day and most commonly between doses when the medication wears off (Nantel and Bronte-Stewart, 2014; Okuma et al., 2018).

FOG management can be divided into pharmacological treatment (Nonnekes et al., 2015) and non-pharmacological treatment, such as exercise (Corcos et al., 2013), deep brain stimulation (Hacker et al., 2020), or cueing devices (Griffin et al., 2011). Meanwhile, due to the limitations and side effects of the pharmacological intervention (Obeso et al., 2000; Aquino and Fox, 2015), more attention has been focused on non-pharmacological interventions, such as resistance exercises can evaluate the severity of FOG and should run through the diagnosis and treatment. The most common evaluation methods include the Timed up and Go test (TUG; Mak and Pang, 2009; Kerr et al., 2010), Unified Parkinson’s Disease Rating Scale (UPDRS; Lun et al., 2005; Kerr et al., 2010), Freezing of Gait Questionnaire (FOG-Q; Giladi et al., 2009; Tambasco et al., 2015) and so on. Nevertheless, most of them have limited specificity and sensitivity for identifying prospective fallers in patients with PD (Boonstra et al., 2008) and may not be sufficiently sensitive to detect changes in balance and walking in the PD population with mild to moderate disease severity (Lo et al., 2010; Fox et al., 2011; Ustinova et al., 2011; Tomlinson et al., 2014).

With the development of wireless communication and microelectronics technology, wearable micro-electro-mechanical systems (MEMS), such as accelerometers and magnetometers, have become small, lightweight and low-cost (Patel et al., 2012). There is a growing interest in using wearable health technology to access FOG and falls. These sensors, generally consisting of accelerometers, gyroscopes, magnetometers and others, can capture body movements in real-time. With a significant advantage compared to clinical scales and conventional assessment tools, the wearable device can act as a personal healthcare worker to help patients evaluate the severity of PD, improve treatment, and avoid the incidence of privacy breaches (Patel et al., 2012; Del Din et al., 2016).

However, owing to the high degree of diversity and complexity of FOG, a huge body of research investigated the feasibility of numerous sensors on various body parts with different algorithms, ranging from machine learning and threshold approaches. There is little agreement on the most effective system design. Meanwhile, most current review articles about FOG detection with wearable sensors ignored the relationship between technology and time. Therefore, we provide a systematic review of the use of wearable systems detect FOG and falls in PD, and the development of this technology, to help guide future research.

## Review methodology

A literature review was performed according to the guidelines of the PRISMA statement. An electronic database search of titles and abstracts was performed by searching Pubmed and Web of Science, and the final search was completed on September 26, 2022. These databases were chosen to allow both medical and engineering journals to be included in the search process. The final search query is summarized in Table 1.

**Table 1:** Search string used for each database.

Database	Search string	Records
PubMed	((((freezing of gait [Title/Abstract]) OR (freezing*[Title/Abstract])) OR (fall*[Title/Abstract])) AND (((wearable*) OR (sensor*)) OR (device*))) AND (Parkinson*[Title/Abstract])	684
Web of Science	(((TI = (sensor*) OR TS = (sensor*) OR TI = (device*) OR TS = (device*) OR TS = (wearable*) OR TI = (wearable*)) AND (TS = (freezing*) OR TI = (freezing*) OR TI = (fall*) OR TS = (fall*)) AND (TI = (Parkinson’s*) OR TS = (Parkinson’s*))))	1,064

The truncation symbol was used to broaden the search with more specificity.

Only original, full-text, peer-reviewed journal articles published in English to access FOG and falls in people with PD were considered in this systematic review. Duplicate findings were removed, and the remaining pieces were relevant according to their title and abstract. Leaving documents were reviewed in full.

Articles were screened based on a series of eligibility standards:

1. Use wearable devices (a single or combination of wearable devices) to collect data as input.
2. Involve people with PD or a dataset of PD.
3. Present original research on the validation of wearable sensors to detect, predict or measure FOG, falls or fall risk.

Studies were excluded:

1. Only examined cueing function for FOG.
2. Only use non-wearable devices to detect or predict FOG or falls.
3. Did not provide sufficient details about the study design and results.

Two reviewers independently screened titles and abstracts included in electronic databases according to eligibility standards. Two reviewers screened the full text of those selected for eligibility. Disagreements between reviewers were resolved by consensus, if needed, after the consultation of a third reviewer. Variable was extracted from chosen research and classified in a predefined table. Authorship, details of the experimental object (i.e., study population, age and medication status), type of sensor, device site, activities, year of publication, evaluation in real-time, the algorithm to process data and classifier performance were all recorded.

## Results

### Studies selection

**Figure 1:**
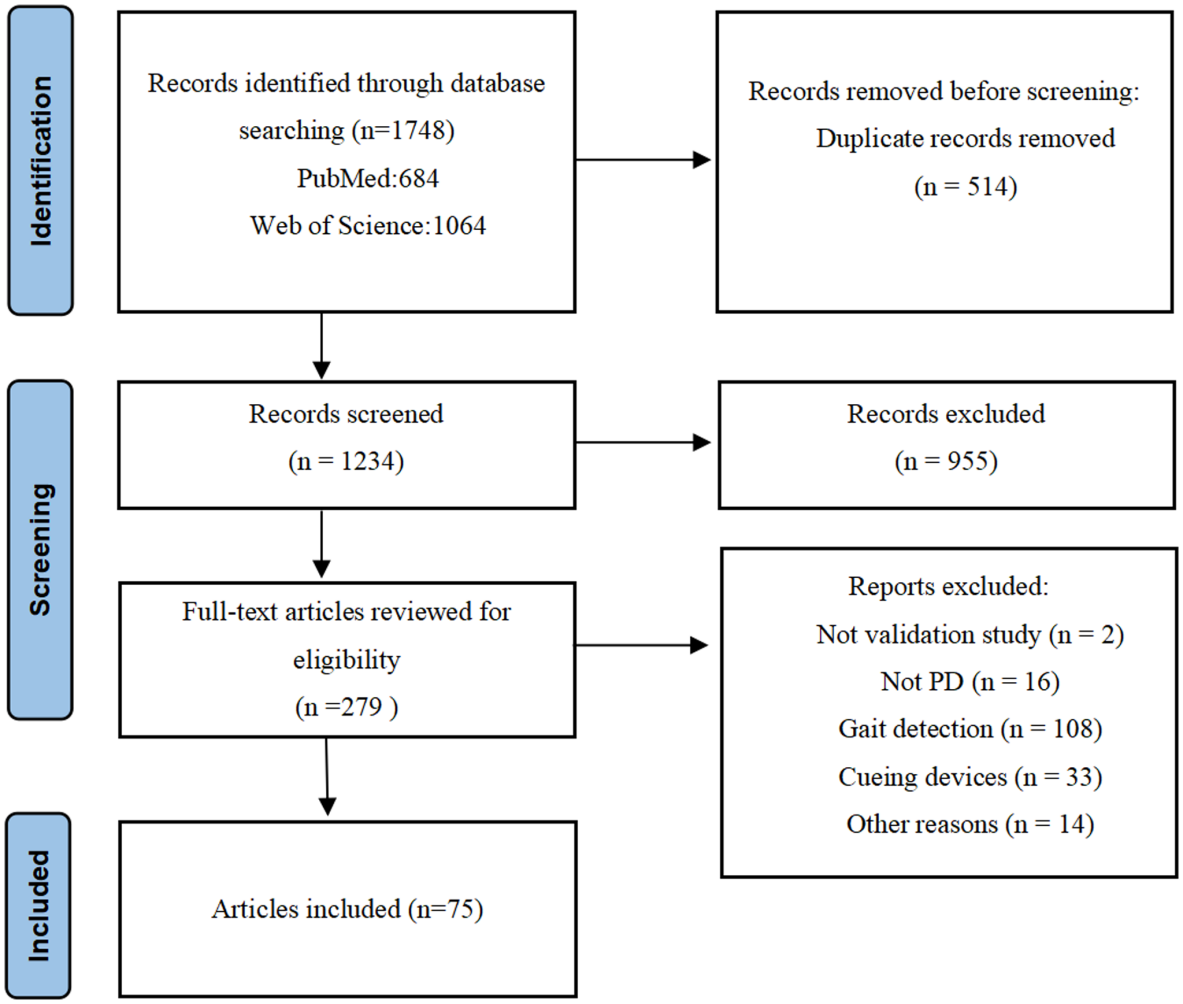
Study flow diagram.

An initial database search identified 1,748 articles that were potentially eligible for inclusion. 514 articles were excluded as duplicates, resulting in 1,234 papers being screened (955 records excluded). The remaining 279 articles were screened by full text. Following screening and eligibility assessment, 75 pieces were shortlisted in this systemic review (72 on FOG detection and 3 on fall detection of PD patients). A complete overview of the selection process is summarized in Figure 1.

### FOG detection

For FOG detection, 72 papers investigated the usage of wearable devices to access FOG in PD (Table 2; Mazilu et al., 2015, 2016; Zach et al., 2015; Capecci et al., 2016; Ahn et al., 2017; Kita et al., 2017; Saad et al., 2017; Camps et al., 2018; Samà et al., 2018; Borzì et al., 2019; Chomiak et al., 2019; Pierleoni et al., 2019; San-Segundo et al., 2019; Ayena and Otis, 2020; Kleanthous et al., 2020; Li et al., 2020; Tang et al., 2020; Dvorani et al., 2021; El-Attar et al., 2021; Esfahani et al., 2021; Ghosh and Banerjee, 2021; Halder et al., 2021; Prado et al., 2021; Shalin et al., 2021; Naghavi and Wade, 2022). The number of subjects used to test the validity of the FOG detection system varied significantly between studies, from 1 (O’day et al., 2020) to 131 (Borzì et al., 2019) (MED = 12). The studied population consisted of patients with Parkinson’s disease, PD patients with diagnosed FOG events (*n* = 35), PD patients with no diagnosed FOG events (*n* = 6), healthy control (*n* = 6) and healthy elderly control (*n* = 1). Furthermore, 43.1% of papers included in this review (*n* = 31) used the data set as a resource to examine the validity of their algorithm. The most commonly used data set was from Bachlin et al. (2010).

**Table 2:** Summary of FOG detection studies.

Author	Studied population	Type of sensor	Device location (n)	Walking task	Algorithm	Classifier (SD)	ON	OFF	Year of publication	Real time	Source of data set
Li et al. (2020)	10 PD	Accelerometer	Thigh (1) Calf (1) Lower back (1)	Walking task	The attention-enhanced LSTM	Sensitivity: 95.1% Specificity: 98.8%	–	–	2020	N	Bachlin et al. (2010)
San-Segundo et al. (2019)	10 PD	Accelerometer	Ankle (1) Thigh (1) Lower back (1)	Walking task and dual task	Random forest, multilayer perceptron and hidden Markov models	Sensitivity 95% Specificity 75%	–	Y	2019	N	Bachlin et al. (2010)
Prado et al. (2021)	10 PD	Pressure sensors Accelerometer Angular velocity Sensor Euler angles sensor	Sole (2)	Zeno Walkway on a standardized 5-m course	Artificial neural network	Sensitivity: 96.0% (2.5) Specificity: 99.6% (0.3) Precision: 89.5% (5.9) Accuracy: 99.5% (0.4)	–	–	2021	Y	?
Mazilu et al. (2016)	18 PD FOG+	Accelerometer Gyroscope	Wrist (2) Ankle (2)	A series of walking task	Supervised machine learning	Subject-dependent accuracy: 85% specificity: 80% Subject-independent Accuracy: 90% Specificity: 66%	Y	–	2016	Y	Mazilu et al. (2013)
Ahn et al. (2017)	10 PD FOG+ 10 HC	Accelerometer Gyroscope Magnetometer	Head (1) Ankle (2)	TUG and 10 m walking task	Threshold	Accuracy: 92.86%	–	Y	2017	Y	–
Tang et al. (2020)	12 PD	Accelerometer Gyroscope	Lower back (1)	TUG	Threshold	Sensitivity: 90.6% (7.71) Specificity: 94.3% (8.36)	–	–	2020	N	–
Borzì et al. (2019)	38 PD FOG+ 93 PD FOG−	Accelerometer Gyroscope Orientation sensor	FOG waist (1) LA thigh (1)	LA test and unscripted and unconstrained activity of daily living	SVM linear, k-NN, neural network and decision tree	LA test AUC: 92% FOG test AUC: 97%	–	–	2019	N	–
Mazilu et al. (2015)	18 PD FOG+	Electrocardiography Skin conductance	Chest (1) Finger (1)	Ziegler protocol, cognitive tasks and hospital tour	Threshold	Predicting accuracy 71.3% with an average of 4.2 s before a freezing episode happened	Y	Y	2015	Y	–
Halder et al. (2021)	10 PD	Accelerometer	Ankle (1) Thigh (1) Hip (1)	Walking task and dual task	k-NN	FOG precision: 95.55% (4.6) sensitivity: 94.97% (4.86) specificity: 99.19% (0.85) F1 score: 95.25% (4.72) accuracy: 98.92% (1.56) Pre of post FOG precision: 92.73% (10.15) sensitivity: 91.5% (10.34) specificity: 99.83% (0.32) F1 score: 92.10% (10.25)	–	–	2021	N	Bachlin et al. (2010)
Zach et al. (2015)	23 PD FOG+	Accelerometer	Waist (1)	Walking task	Threshold	Full rapid turns sensitivity: 78% specificity: 59% Walking rapidly with small steps sensitivity: 64% specificity: 69% Combining all tasks sensitivity: 75% specificity: 76%	–	Y	2015	N	–
Camps et al. (2018)	21 PD	Accelerometer Gyroscope Magnetometer	Waist (1)	Walking task and dual task	Deep learning eight-layered 1D-ConvNet	Accuracy: 89% Sensitivity: 91.9% Specificity: 89.5%	Y	Y	2018	N	REMPARK project
el-Attar et al. (2021)	10 PD	Accelerometer	Ankle (1) Knee (1) Hip (1)	Walking task and dual task	SVM and artificial neural network	SVM accuracy: 87.5% Neural network accuracy: 93.8%	–	–	2021	N	Bachlin et al. (2010)
Capecci et al. (2016)	20 PD FOG+	Accelerometer	Hip (1)	TUG and dual task	Threshold	Moore-Bächlin Algorithm sensitivity: 70.1% specificity: 84.1% Moore-Bächlin Algorithm with step cadence sensitivity: 87.57% specificity: 94.97%	–	–	2016	Y	–
Naghavi and Wade (2022)	7 PD	Accelerometer Gyroscope	Ankle (2)	Walking task	Convolutional neural network, transfer learning and k-means clustering	Sensitivity: 63.0% Specificity: 98.6% Target models identified 87.4% FOG on sets, with 21.9% predicted	–	–	2022	Y	Naghavi et al. (2019)
Saad et al. (2017)	5 PD	Accelerometer Telemeter Goniometer	Shin (1)	Walking task	Gaussian neural network	Efficiency: 87%	–	–	2017	N	–
Pierleoni et al. (2019)	10 PD	Accelerometer Gyroscope Magnetometer	Chest (1)	Walking task	Threshold	Accuracy: 99.7%	–	–	2019	Y	–
Samà et al. (2018)	15 PD	Accelerometer	Waist (1)	Walking task and dual task	Threshold	Sensitivity: 91.7% Specificity: 87.4%	Y	Y	2018	Y	MASPARK project
Ghosh and Banerjee (2021)	10 PD	Accelerometer	Leg (2) Hip (2)	Walking task and dual task	Linear discriminant analysis, classification and regression trees, SVM and random forest.	Accuracy: 89.94% Sensitivity: 87.8% Specificity: 93.02%	–	–	2021	N	Bachlin et al. (2010)
Kita et al. (2017)	32 PD	Accelerometer Gyroscope	Shin (2)	Walking task	Threshold	Specificity 97.57% Sensitivity 93.41% Precision 89.55% Accuracy 97.56%	–	–	2017	N	–
Esfahani et al. (2021)	10 PD	Accelerometer Gyroscope Magnetometer	Shank (1) Thigh (1) Lower back (1)	Walking task	LSTM	Sensitivity 92.57% Specificity 95.62%	–	–	2021	N	Bachlin et al. (2010)
Kleanthous et al. (2020)	10 PD FOG+	Accelerometer	Ankle (1) Thigh (1) Trunk (1)	Walking task	Random forest, extreme Gradient boosting, Gradient boosting, SVM using radial basis functions, and neural network	SVM FOG sensitivity: 72.34% specificity: 87.36% Transition sensitivity: 91.49% specificity: 88.51% Normal activity sensitivity: 75% specificity: 93.62%	–	Y	2020	N	Bachlin et al. (2010)
Ayena and Otis (2020)	12 PD 9 HEC 10 HC	Force sensitive resistor Accelerometer	Sole (2)	TUG	Threshold	A significant difference was found for three FSR and IMU and on FSR and IMU in the elderly population (*p* < 0.001)	–	–	2020	N	–
Shalin et al. (2021)	11 PD FOG+	Accelerometer Plantar pressure sensor	Sole (2)	Walking task	LSTM	Sensitivity: 82.1% (6.2) Specificity: 89.5% (3.6)	–	–	2021	Y	–
Dvorani et al. (2021)	16 PD FOG+	Accelerometer	Shoe (2)	Walking task	SVM and Adaboost classifiers	Sensitivity: 88.5% (5.8) Specificity: 83.3% (17.1) AUC: 92.8% (5.9)	–	–	2021	Y	?
Chomiak et al. (2019)	21 PD 9 HC	Accelerometer Gyroscope	Above the patellofemoral joint line (1)	Walking task and dual task	Nonlinear m-dimensional phase-space data extraction and Monte Carlo analysis	Error rate: 0% Sensitivity: 100% Specificity: 100%	–	–	2019	Y	–
Borzì et al. (2021)	11 PD FOG+	Accelerometer Magnetometer Gyroscope	Shin (2)	TUG standardized 7-m course	Linear discriminant analysis and SVM	The implemented classification algorithm in patients on (off) therapy sensitivity: 84.1% (85.5%), specificity: 85.9% (86.3%) accuracy: 85.5% (86.1%) Machine learning sensitivity: 84.0% (56.6%) specificity: 88.3% (92.5%) accuracy: 87.4% (86.3%)	Y	Y	2021	Y	–
Kim et al. (2018)	32PD	Accelerometer Gyroscope	In the trouser pocket (1)	A series of walking tasks	Convolutional neural network	Average sensitivity of 93.8% and a specificity of 90.1%	–	–	2018	N	–
Marcante et al. (2021)	20 PD	Accelerometer Plantar pressure sensors	Sole (2)	A series of walking tasks	Threshold	Accuracy: 90% False positive rate: 6% False negative rate: 4%	Y	Y	2020	N	–
Mancini et al. (2021)	Study I: 27 PD FOG+ 18 PD FOG− Study II: 23 PD FOG+ 25 PD FOG−	Accelerometer Gyroscope Magnetometer	Study I: Shin (2) Foot (2) Wrist (2) Sternum and posterior trunk over L5 (1) Study II: Foot (2) over the lumbar area (1)	Walking task	Open-source algorithm	Rater 1 accuracy: 88% sensitivity: 89% specificity: 88% false positive rate: 13% false negative rate: 11% AUC: 93% Rater 2 accuracy: 85% sensitivity: 80% specificity: 87% false positive rate: 13% false negative rate: 20% AUC: 89%	–	Y	2021	N	–
Pardoel et al. (2021)	11 PD	Accelerometer Gyroscope Plantar pressure sensor	Sole (2) Shank (2)	A series of walking task	Decision tree ensemble model	1 s window classification of Total-FOG data sensitivity: 76.4% specificity: 86.2% The transition between Pre-FOG gait and FOG sensitivity: 85.2% The FOG window sensitivity: 93.4%	–	Y	2021	Y	–
Prateek et al. (2018)	16 PD	Accelerometer Gyroscope	The heel of shoe (2)	A series of walking task	Threshold	Accuracy: 81.03%	–	–	2018	N	–
Bikias et al. (2021)	11 PD	Accelerometer Gyroscope	Wrist (1)	–	Machine learning	Leave-one-subject-out cross-validation sensitivity: 83% specificity: 88% fold cross-validation schemes sensitivity: 86% specificity: 90%	–	–	2021	N	–
Naghavi and Wade (2019)	10 PD	Accelerometer	Shank (1) Thigh (1) Lower back (2)	Two walking tasks and one dual task	Threshold	Accuracy: 88.8% Sensitivity: 92.5% Specificity: 89.0%	–	Y	2019	Y	Bachlin et al. (2010)
Pardoel et al. (2021)	11 PD FOG+	Accelerometer Gyroscope	Knee (2) Ankle (2)	Walking task along a complex pathway to provoke FOG	Threshold	Detection model episodes identified: 92.1% (8.2%) precision: 31.8% (19.9%) Prediction model episodes identified: 93.8% (6.8%) precision: 30.6% (17.0%)	Y	–	2021	N	–
Mesin et al. (2022)	12 PD FOG+	Accelerometer Gyroscope Electroencephalogram Skin conductance Electromyography Electrocardiogram	Lateral tibia of the leg (2) Fifth lumbar spine (1) Wrist (1)	A series of walking task	SVM and k-NN	Subject-independent accuracy: 85% subject-dependent accuracy: 88%	–	Y	2022	N	Zhang et al. (2022)
Demrozi et al. (2020)	10 PD	Accelerometer	Back (1) Hip (1) Ankle (1)	Walking task	k-NN	Sensitivity: 94.1% Specificity: 97.1%	–	–	2020	Y	Bachlin et al. (2010)
Mikos et al. (2019)	25 PD	IMU	Ankle (2)	TUG standardized 7-m course	Neural network	Sensitivity: 95.9% Specificity: 93.1%	–	–	2019	Y	?
Reches et al. (2020)	71 PD FOG+	Accelerometer Gyroscope Magnetometer	Lower back (2) Ankle (2)	A series of walking tasks and dual task	SVM with the radial basis function	Sensitivity: 84.1% Specificity: 83.4% Accuracy: 85.0%	Y	Y	2020	N	?
Sigcha et al. (2020)	21 PD FOG+	Accelerometer	Waist (1)	20 min of scripted ADL	Recurrent neural network	Mean sensitivity: 87.1% Mean specificity: 87.1% Mean AUC: 93.9%	–	–	2020	N	Rodríguez-Martín et al. (2017)
Ahlrichs et al. (2016)	8 PD FOG+ 12 PD FOG−	Accelerometer Gyroscope Magnetometer	–	Scripted activities simulating natural behavior at the patients’ home	SVM	Sensitivity:92.3% Specificity:100%	–	–	2016	Y	Rodriguez-Martin et al. (2015)
Pham et al. (2017)	10 PD	Accelerometer	Shank (1) Thigh (1) Lower back (1)	Walking task	Anomaly score detector with adaptive thresholding	Sensitivity: 96% Specificity: 79% Ankle only accuracy: 94% specificity: 84% Lower back only accuracy: 89% specificity: 94%	–	Y	2017	N	Bachlin et al. (2010)
Suppa et al. (2017)	28 PD FOG+ 16 PD FOG−	Accelerometer Gyroscope	Shin (2)	TUG on standardized 3-m course	*Ad hoc* algorithms	Accuracy: 98.51% Sensitivity: 93.41% Specificity: 98.51% Positive predictive: 89.55% Negative predictive: 97.31%	Y	Y	2017	N	–
Ren et al. (2022)	12 PD FOG+	Accelerometer Gyroscope Force sensing resistor sensors	Waist (1) Thigh (2) Shank (2) Sole (2)	Walking task	Threshold	Left shank gyro and accelerometer sensitivity 78.39% specificity 91.66% accuracy 88.09 precision 77.58% f-score 77.98%	Y	–	2022	N	?
Ashfaque Mostafa et al. (2021)	10 PD FOG+	Accelerometer	Shank (1) Thigh (1) Lower back (1)	Unscripted and unconstrained activities of daily living in an apartment-like setting	Moving windows extracted from the signals, handcrafted features, recurrence plots, short-time Fourier transform, discreet wavelet transform, Pseudo Wigner Ville distribution with deep learning-based LSTM and convolutional neural networks	Window size of 3 accuracy: 98.5% sensitivity: 98.5% specificity: 97.9% Window size of 4 sensitivity: 96.9% specificity: 96.7%	–	–	2021	N	Bachlin et al. (2010)
Guo et al. (2019)	10 PD	Accelerometer	Ankle (1) Thigh (1) Hip (1)	Walking task and dual task	The time-varying autoregressive moving average model	Sensitivity: 99.2% Specificity: 94.59% Accuracy average of sensitivity: 96.86% specificity: 96.9%	–	Y	2019	N	Bachlin et al. (2010)
Azevedo Coste et al. (2014)	4 PD	Accelerometer Gyroscope Magnetometer	Shank (1)	Walking task with dual tasking	Threshold	Sensitivity: 79.5% Specificity: not reported Only number of falls positives: 13 vs.35 true positives	–	–	2014	N	–
Naghavi et al. (2019)	18 PD	Accelerometer	Ankle (2)	A series of daily walking tasks	ADAptive SYNthetic sampling algorithm	Accuracy: 97.4% Prediction: 66.7%	–	–	2019	Y	Schaafsma et al. (2003)
O’day et al. (2020)	1 PD FOG+	IMU	Shank (2)	Walking task	Closed-loop DBS algorithms	–	–	–	2019	Y	–
Kim et al. (2015)	15 PD FOG+	Accelerometer Gyroscope	Waist (1) Trouser pocket (1) Shin (1)	Walking task and dual (single) task	AdaBoost.M1 classifier	Waist only sensitivity: 86% specificity: 91.7% Trouser pocket only sensitivity: 84% specificity: 92.5%	–	–	2015	N	–
Palmerini et al. (2017)	18 PD	Electrocardiography Skin-conductance	Shank (2) Lower back (1)	Walking task and dual task	Threshold	AUC: 76% Sensitivity: 83% Specificity: 67%	Y	–	2017	Y	Mazilu et al. (2015)
Cole et al. (2011)	10 PD 2 HC	Accelerometer Electromyographic	Forearm accelerometer (1) Thigh accelerometer (1) Skin accelerometer and Electromyographic (1)	Unscripted and unconstrained activities of daily living in an apartment-like setting	Dynamic neural network and linear classifier	Sensitivity: 82.9% Specificity: 97.3%	–	–	2011	N	–
Rezvanian and Lockhart (2016)	10 PD FOG+	Accelerometer	Shank (1) Thigh (1) Lower back (1)	A series of walking task	Continuous wavelet transform	Skin only sensitivity: 84.9% specificity: 81.0% Thigh only sensitivity: 73.6% specificity: 79.6% Lower back only: sensitivity: 83.5% specificity: 67.2%	Y	Y	2016	N	Bachlin et al. (2010)
Pardoel et al. (2022)	11 PD FOG+	Plantar pressure sensor	Sole (2)	Walking task and dual task	Decision tree and random undersampling boosting	Sensitivity: 77.3% Specificity: 82.9%	–	–	2022	N	Pardoel et al. (2021)
Tripoliti et al. (2013)	5 PD FOG+ 6 PD FOG− 5 HC	Accelerometer Gyroscope	Wrist (2) Shin (2) Waist (1) Chest (1)	A series of walking tasks	Threshold	Sensitivity: 81.94% Specificity: 98.74%	Y	Y	2013	N	–
Aich et al. (2018)	36 PD FOG+ 15 PD FOG−	Accelerometer	Knee (2)	Walking task	Naïve Bayes, k-NN, SVM and decision tree	Accuracy: 89.139% Sensitivity: 88.524% Specificity: 88.769%	–	–	2018	N	–
Arami et al. (2019)	10 PD FOG+	Accelerometer	Lower back (1) Thigh (2) Shank (2)	Walking task	SVM and probabilistic neural networks	Sensitivity: 93% (4) Specificity: 91% (6)	Y	–	2019	Y	Bachlin et al. (2010)
Guo et al. (2022)	12 PD FOG+	Electroencephalography	Waist on L5 (1) Leg (2)	Two TUG tasks	LSTM	Cross-subject setting GM: 91.0% (3.5%) Subject-dependent setting GM: 91.0% (5.0%)	–	Y	2022	N	–
Moore et al. (2013)	25 PD	Accelerometer	Lumbar region of the back (1) Thigh (2) Shank (2) Foot (2)	TUG tasks	Threshold	Lower back sensor, 10s window: sensitivity: 86.2% specificity: 82.4%	–	Y	2013	N	–
Moore et al. (2007)	11 PD FOG+ 10 HC	Accelerometer	Shank (1)	A series of walking task	Threshold	Accuracy: 89% Sensitivity: 89% False positives: 10%	Y	Y	2008	N	–
Mazzetta et al. (2019)	7 PD FOG+	Accelerometer Gyroscope Electromyography	Tibialis anterior (1) Gastrocnemius of the right leg (1)	TUG on standardized 7-m course	Threshold	False negative: 2% False positive: 5%	Y	Y	2019	Y	–
Rodríguez-Martín et al. (2017)	21 PD FOG+	Accelerometer	Waist (1)	A set of scripted activities at patients’ home	SVM	Generic model sensitivity: 74.7% specificity: 79.0% Personalized model sensitivity: 88.09% specificity: 80.09%	Y	Y	2017	Y	REMPARK project
Shi et al. (2022)	63 PD FOG+	Accelerometer Gyroscope Magnetometer	Ankle (2) 7th cervical vertebra (1)	TUG on standardized 7-m course and daily routine	Continuous wavelet transform and convolutional neural network	Geometric mean: 90.7% F1 score: 91.5%	–	–	2022	N	–
Kwon et al. (2014)	20 PD FOG+	Accelerometer	Shoe (1)	A walking task	Threshold	Sensitivity: 86% Specificity: 86%	Y	–	2014	N	–
O’Day et al. (2022)	16 PD	IMU	Chest (1) Lumbar region (1) Ankle (2) Feet (2)	Free-living setting	Convolutional neural network	Lumbar and both ankles AUROC: 83%	–	Y	2022	N	–
Shi et al. (2020)	67 PD FOG+	Accelerometer Gyroscope Magnetometer	Lateral malleolus area of the ankles (2) 7th cervical vertebra of the spine (1)	TUG on standardized 7-m course	Convolutional neural network and continuous wavelet transform	Accuracy: 89.2% Geometric mean: 88.8%	–	Y	2020	N	–
Yungher et al. (2014)	14 PD FOG+	Accelerometer Gyroscope Magnetometer	Lower back (1) Thigh (2) Shin (2) Foot (2)	TUG on standardized 5-m course	Threshold	–	–	Y	2014	N	–
Ly et al. (2017)	6 PD FOG+	Electroencephalography	Head (1)	A series of TUG	Bayesian Neural Networks and time-frequency Stockwell Transform	Sensitivity: 84.2% Specificity: 88% Accuracy: 86.2%	–	Y	2017	N	–
Jovanov et al. (2009)	1 PD 4 non-PD	Accelerometer Gyroscope	Knee (1)	Walking task	Threshold	The average detection latency for five experiments was 332 ms and the maximum latency was 580 ms.	–	–	2009	Y	–
Tzallas et al. (2014)	Lab 24 PD FOG Home 12 PD FOG	Accelerometer Gyroscope	Wrist (2) Skin (2) Waist (1)	Lab: a series of walking tasks. Home: 5 consecutive days of free living.	Hidden Markov Model and SVM	Lab accuracy: 79% Home mean absolute error: 79%	Y	Y	2014	N	–
Stamatakis et al. (2011)	1 PD 1 HC	Accelerometer	Hallux Heel (1) Foot (2)	Walking task	Threshold	–	–	–	2011	N	–
Rodríguez-Martín et al. (2017)	12 PD	Accelerometer Gyroscope	Waist (1)	Walking task, dual-task and free-living setting for 3 days	SVM	Sensitivity: 82.08% Specificity: 93.75%	Y	Y	2017	Y	–
Iakovakis et al. (2016)	5 PD 10 HC	Sphygmomanometer Smartwatch	Wrist (2)	Walking task	SVM, linear regression and neural network	Linear regression predictive accuracy: 73%	–	–	2016	Y	–

PD, Parkinson’s disease; FOG, freezing of gait; FOG+, PD patients with diagnosed FOG events; FOG−, PD patients with no diagnosed FOG events; HC, healthy control; HEC, health elderly control; LA, leg agility; k-NN, k-nearest neighbor; SVM, support vector machine; LSTM, long short term memory; FSR, force sensitive resistor; IMU, inertial measurement unit; TUG, time up and go test; AUC, area under the curve; ADL, activity of daily living; ON, subjects were in the ON medication state; OFF, subjects were in the OFF medication state; REMPARK, Remote and Autonomous Management of Parkinson’s Disease; MASPARK, Improving Quality of Life with an Automatic Control System, a question mark means articles used data set but did not provide the source of data set or cannot be found.

**Figure 2:**
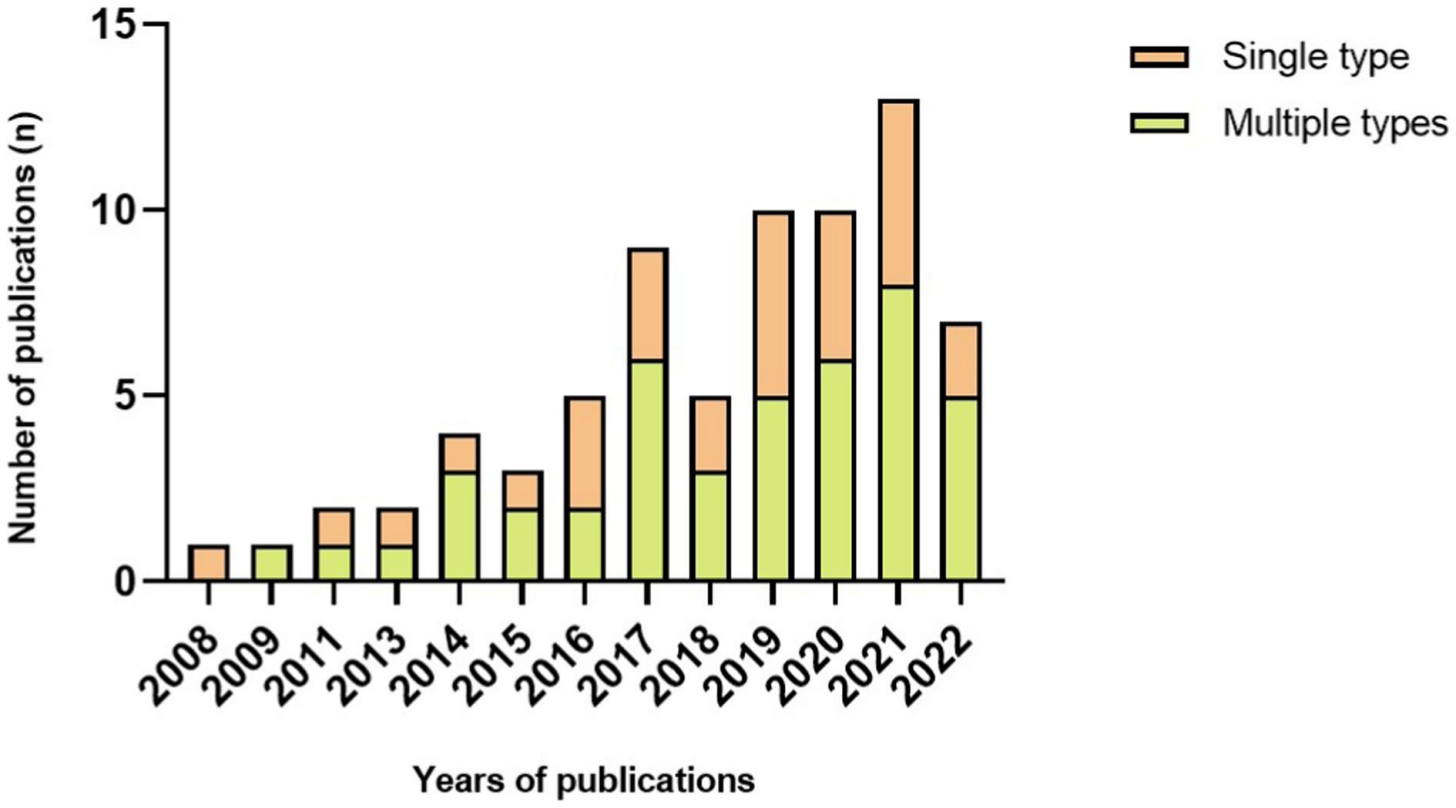
Number of publications each year per number of sensor type.

**Figure 3:**
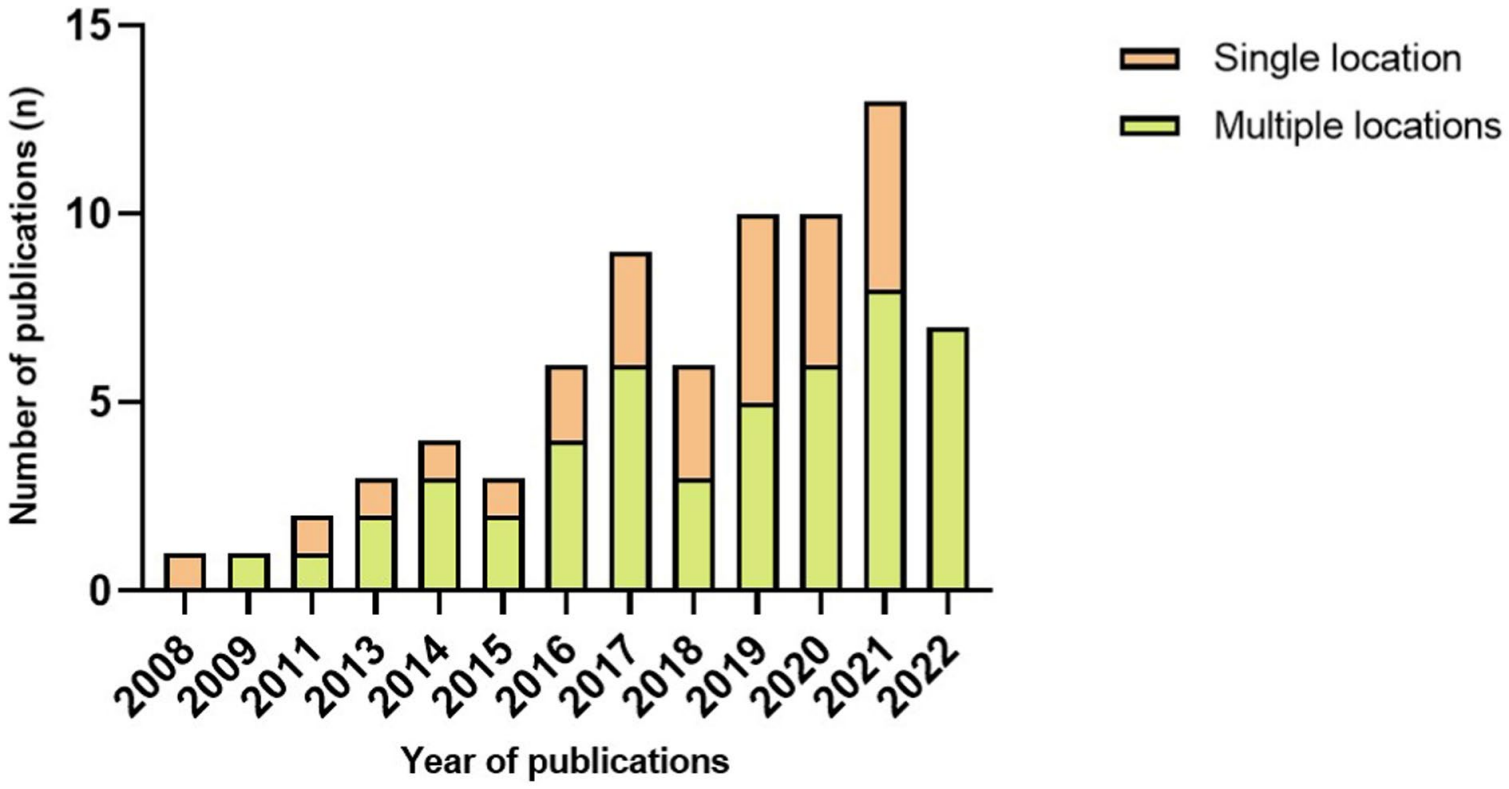
Number of publications each year per number of sensor locations.

Device type and placement are remarkably diverse between studies. Concerning the type of sensor, 27 papers used a single type of wearable device to implement FOG detection, including 25 articles that used an accelerometer, two with electroencephalography and one with plantar pressure sensors. It is important to note that 45 articles used multiple wearable device types to access FOG detection (Figure 2). The combination of accelerometers and gyroscopes was the choice of 15 papers, and 12 pieces combined accelerometers, gyroscopes and magnetometers to access FOG detection. Likewise, wearable devices are located on various parts of the human body. Of the 72 included studies, the same number of papers reported placing a wearable device on the thigh and ankle (22.22% of studies, n = 16, 3 times as the single site on the ankle), the shank (19.44% of studies, *n* = 14, 4 times as the single location), the waist (16.67% of studies, *n* = 12) and the lower back (16.67% of studies, *n* = 12, 6 times as the single location). Details on the studies included in this systematic review that reported placement are summarized in Figure 3 and Table 3.

**Table 3:** Summary of device location of FOG detection studies.

Body part	Body landmark or placement	Number of articles (n)	Ratio (%)	Single location (n)
Head and neck	Head	2	2.78	1
	7th cervical vertebra	2	2.78	0
Upper limb	Forearm	1	1.39	0
	Wrist	7	9.72	1
	Finger	1	1.39	0
Trunk	Chest	4	5.56	1
	Back	1	1.39	0
	Lower back	12	16.67	1
	Lumbar	4	5.56	0
	Trunk	1	1.39	0
	Waist	12	16.67	6
Lower limb	Foot	4	5.56	0
	Calf	1	1.39	0
	Gastrocnemius	1	1.39	0
	Hallux	1	1.39	0
	Heel	2	2.78	0
	Hip	6	8.33	1
	Knee	4	5.56	2
	Lateral tibia of leg	1	1.39	0
	Leg	2	2.78	0
	Sole	7	9.72	5
	Shank	13	18.06	4
	Shin	8	11.11	3
	Shoe	2	2.78	2
	Thigh	16	22.22	0
	Tibialis anterior	1	1.39	0
	Trouser pocket	2	2.78	1
	Ankle	16	22.22	3

**Figure 4:**
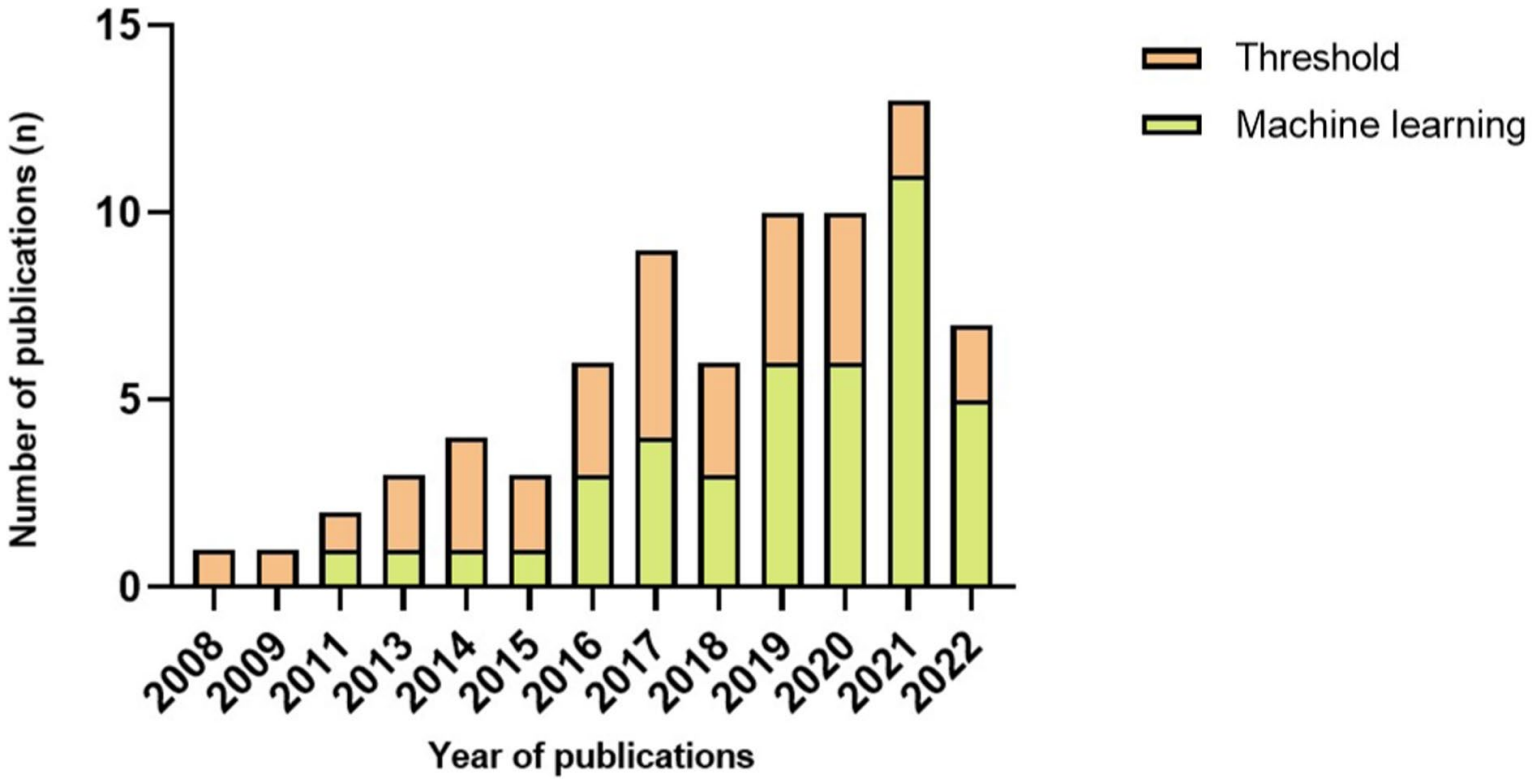
Number of publications each year per type of algorithm.

The algorithm plays a vital role in FOG detection and varies in complexity. Generally, it can be categorized into threshold and machine learning. Of 72 papers, 30 used threshold-based algorithms to detect FOG, leaving 42 pieces used machine learning. In Figure 4, we observed that the number of articles that used thresholds was more than or equal to articles that used machine learning before 2019. Since then, more papers have used machine learning than the threshold, even five times higher in 2021. Evaluation in real-time was the choice of 24 articles. Machine learning algorithms were used in 15 of the 24 articles, leaving 9 papers that used threshold algorithms to detect a FOG episode as it occurs.

Among the 73 articles investigating FOG detection, a vast majority of studies (*n* = 71) reported measures of validation performance [e.g., sensitivity, specificity, accuracy, area under the curve (AUC) or f-score], and 2 studies did not report validity measures (Stamatakis et al., 2011; Yungher et al., 2014). Overall, the sensitivities reported in the reviewed studies ranged from 63 to 100%, from 59 to 100% for specificity, from 71.3 to 99.7% for accuracy, AUC ranged from 76 to 97% and f-score ranged from 77.98 to 92.10% (Table 4).

**Table 4:** Number of publications per type of outcome for each sensor combination.

Combination	Number of articles (n)	Ratio (%)	Sensitivity	Specificity
Accelerometer and gyroscope	15	34.1	63–100% (MED = 86%)	66–100% (MED = 92.9%)
Accelerometer, gyroscope and magnetometer	12	27.3	56.6%−92.6 (MED = 84.1%)	83.4–100% (MED = 88.2%)
Pressure sensor, accelerometer, angular velocity sensor and Euler angles sensor	1	2.3	96%	99.6%
Accelerometer, gyroscope and orientation sensor	1	2.3	–	–
Electrocardiography and skin conductance	2	4.5	83%	67%
Accelerometer, telemeter and goniometer	1	2.3	–	–
Accelerometer and force sensor	3	6.8	82.1%	89.5%
Accelerometer, gyroscope and force sensor	2	4.5	76.4–93.4% (MED = 84.9)	86.2–91.66% (MED = 88.9)
Accelerometer, gyroscope, electroencephalogram, skin conductance, electromyography and electrocardiogram	1	2.3	–	–
IMU	3	6.8	94.1%	97.1%
Accelerometer and electromyographic	1	2.3	82.9%	97.3%
Accelerometer, gyroscope and electromyography	1	2.3	–	–
Sphygmomanometer and smartwatch	1	2.3	–	–

### Fall detection

A total of 3 papers on fall detection were included and varied in the study population, approach and performance (Table 5). The number of subjects ranged from 12 to 29 (MED = 15), and the studied population can be categorized into patients with PD (*n* = 3), healthy control (*n* = 1) and healthy elderly control (*n* = 1). None of them used a data set.

**Table 5:** Summary of fall detection studies.

Author	Studied population	Type of sensor	Device location	Walking task	Algorithm	Classifier	ON	OFF	Year of publication	Real time	Source of data set
Greene et al. (2018)	15 PD	Accelerometer Gyroscope	Shank (2)	The free-living setting for 6 months	Threshold	Accuracy 73.33%	–	–	2018	N	–
Takač et al. (2013)	12 PD	Accelerometer Gyroscope	Waist (1)	Walking task performed	Neural network	root mean square error (RMSE) = 0.16	–	–	2013	Y	–
Ayena et al. (2016)	7 PD 12 Young non-PD 10 Elderly non-PD	Accelerometer Force sensor Bending sensor	Sole (2)	Participants performed the OLST at home as part of a serious game for balance training	Threshold	The proposed OLST score was not significantly different from the iOLST score in all groups. Discriminant validity-Proposed OLST score was significantly different between PD and non-PD subjects. The proposed OLST score has significantly differed between ground types	Y	–	2016	Y	–

PD, Parkinson’s disease; ON, subjects were in the ON medication state; OFF, subjects were in the OFF medication state; OLST, one-leg standing test.

All articles used multiple wearable devices. However, the type of sensor and placement are remarkably diverse between studies. Two pieces used accelerometers and gyroscopes to detect falls, while the remaining one used an accelerometer, force sensor and bending sensor. As for device location, 1 article placed sensors on the shank, 1 on the waist and 1 on the insole. Regarding the algorithm, 2 papers used threshold to process data, leaving 1 article used machine learning. Three articles reported fall detection performance, but only two performed fall detection in real-time. Meanwhile, the measure of validation performance was varied. One piece used accuracy (73.33%), and one used root mean square error (0.16), leaving one article mentioning the data difference.

## Discussion

This systematic review aimed to examine the articles of FOG and fall detection area to determine the best type of wearable devices, the most appropriate device locations, and the most effective approaches to processing data, which can balance accuracy and immediacy. This paper also discussed the recent trend of related technologies. A total of 75 articles were included in this review, 72 on FOG and 3 on falls.

### FOG/falls detection apparatus

The apparatus used in FOG or fall detection can be generally divided into wearable devices and context-aware systems. Due to the development of wireless communication and microelectronics technology, many researchers focus on wearable devices to detect FOG or falls. In this review, the type of sensors and the combination are remarkably diverse between studies. Twenty-eight studies used a single type of wearable device to detect FOG, and 92.9% of them relied on accelerometers only (*n* = 26), and the sensitivity of using an accelerometer only ranged from 70.1 to 99.2% (MED = 88.52%), and the specificity ranged from 59 to 99.83% (MED = 88%). Meanwhile, 2 studies used electroencephalography only, while Pardoel et al. (2022), the pressure sensor was the only device for FOG detection, its sensitivity ranged from 77.3 to 84.2%, and specificity ranged from 82.9 to 88%. These results indicated that the type of sensor would not affect the accuracy of using a single type of sensor. The use of single kind of sensor can reduce the calculation and complexity of the FOG detection system.

In this review, we found a large proportion of studies using IMU, which often consists of more than one type of sensor, have become popular in FOG and fall detection applications. As shown in Table 2, a total of 44 papers utilized IMU for FOG detection, 3 of them only mentioned IMU, remaining 41 articles illustrated the type of sensors. The combination consisting of an accelerometer and a gyroscope was the most popular in this review, 15 papers used this combination, and the combination of an accelerometer, a gyroscope and a magnetometer was the choice of 12 articles. The difference in validation performance (e.g., sensitivity and specificity) between combinations were slight, except for the specificity of the combination of electrocardiography and skin conductance (67%). Multiple types of sensors were the choice of 3 articles to detect falls in patients with PD. There might be several reasons behind this trend. First, the IMUs can provide multidimensional data to measure body movement of FOG and fall detection, improving the validation performance. Second, the rapid development of MEMS facilitated lower energy consumption and small-sized chips with low cost, which makes the placement of wearable IMUs much easier. Third, as machine-learning technology advances rapidly, researchers can process vast quantities of data and conclude with high accuracy.

### Device location

As mentioned above, various protocols were described concerning the device’s location on the human body to detect FOG and falls. Generally, the human body is divided into the head and neck, trunk, upper limb and lower limb. Of the 72 studies included in this review, 84.7% studies used the lower limb as a wearable device location (*n* = 61). The most popular placements were the thigh (*n* = 16) and the ankle (*n* = 16). Besides, the sole was the most common single placement on the lower limb. The results also showed that the waist (*n* = 12) and lower back (*n* = 12) were the most used on the trunk, and the waist (*n* = 6) was the most frequent single placement on the human body. Considering fall detection, 2 articles used the lower limb as a wearable sensor location, leaving 1 article placed sensors on the upper limb. The critical task of the lower limb is to support the entire body. Changes in the lower limb (e.g., velocity, direction and speed) can intuitively reflect the status of patients.

### FOG/fall detection algorithms

FOG and fall detection approaches vary in complexity. Threshold-based algorithm appeared to be the most straightforward method in FOG and fall detection. A total of 30 articles included in this review use threshold-based algorithms in FOG detection. As for fall detection, 2 papers used threshold-based algorithms. With threshold-based algorithms, the occurrence of FOG and falls are considered to be detected if indicators are beyond a specific threshold. Otherwise, the event of FOG/fall does not exist. With the advantage of being computationally efficient, threshold methods can process data in a short period, making them easily used in real-time systems. However, the drawback of the threshold-based algorithm is obvious. Generally, a high threshold may lead to a low false positive rate but also ignore some occurrences of FOG/fall, and vice versa. This is the conundrum that almost current researchers have to face.

To improve the accuracy of FOG and fall detection, machine learning algorithms, including SVM (Tzallas et al., 2014; Ahlrichs et al., 2016; Iakovakis et al., 2016; Rodríguez-Martín et al., 2017; Aich et al., 2018; Arami et al., 2019; Borzì et al., 2019, 2021; Kleanthous et al., 2020; Reches et al., 2020; Dvorani et al., 2021; El-Attar et al., 2021; Ghosh and Banerjee, 2021; Mesin et al., 2022), k-NN (Aich et al., 2018; Borzì et al., 2019; Demrozi et al., 2020; Halder et al., 2021; Mesin et al., 2022), decision trees (Aich et al., 2018; Borzì et al., 2019; Pardoel et al., 2021, 2022), hidden Markov model (Tzallas et al., 2014; San-Segundo et al., 2019), neural network (Cole et al., 2011; Iakovakis et al., 2016; Ly et al., 2017; Saad et al., 2017; Kim et al., 2018; Arami et al., 2019; Borzì et al., 2019; Mikos et al., 2019; Kleanthous et al., 2020; O’day et al., 2020, 2022; Shi et al., 2020, 2022; Sigcha et al., 2020; Ashfaque Mostafa et al., 2021; El-Attar et al., 2021; Prado et al., 2021; Naghavi and Wade, 2022), random forest (San-Segundo et al., 2019; Kleanthous et al., 2020; Ghosh and Banerjee, 2021) and LSTM (Li et al., 2020; Ashfaque Mostafa et al., 2021; Esfahani et al., 2021; Shalin et al., 2021; Guo et al., 2022), were used extensively in recent studies. Data were collected from sensors, and a training period is necessary for machine learning. Machine learning can improve the validation performance of FOG/fall detection but might require a longer time for data processing. With the development of computer technology, studies have increasingly examined machine learning algorithms in real-time FOG detection. Furthermore, the utilization of machine learning algorithms to identify FOG is becoming the primary current for the sake of improving validation performance (Figure 3).

### FOG/fall detection performance

The validation performance of FOG/fall detection varies, including sensitivity, specificity, accuracy, AUC and f-score. Among the 73 articles investigating FOG detection, the sensitivities ranged from 63 to 100%. The highest sensitivity (100%) was achieved by Chomiak et al. (2019) and the lowest sensitivity (63%) was reported by Naghavi et al. (2019). The specificities were from 59 to 100%. The lowest specificity (59%) was written by Zach et al. (2015) and only one article reported 100% specificity (Chomiak et al., 2019). Some papers used accuracy as a validation performance standard, ranging from 71.3 to 99.7%. The accuracy (71.3%) in Mazilu et al. (2015) was the lowest, and the highest accuracy (99.7%) was achieved by Pierleoni et al. (2019). A few studies reported AUC ranged from 76 to 97%. The highest AUC (97%) was achieved by Borzì et al. (2019) and the lowest AUC (76%) was in Palmerini et al. (2017) A few studies utilized f-score to evaluate validation performance ranging from 77.98 to 92.10%. The lowest f-score (77.98%) was reported by Ren et al. (2022) and the highest f-score (92.10%) was written by Halder et al. (2021). Meanwhile, the measure of validation performance various considerably, including accuracy (73.33%, *n* = 1), root mean square error (0.16, *n* = 1) and data difference (*n* = 1).

It should be noted that the conclusion of the best FOG/fall detection based on the reported validation performance is unwarranted since the collection approaches of FOG/fall data varies considerably, including methods of provoking FOG/fall, and the number of subjects varied, which might affect the validation performance.

## Conclusion

Based on 75 articles on wearable device utilization for FOG and fall detection in patients with PD, this review represented the recent trend and several critical aspects in current research, including the type of sensors, device location, FOG/fall algorithms, the number of subjects (or data set) and validation performance. Research on FOG and fall detection has been developed rapidly in recent years, and emerging technology like machine learning can balance accuracy and immediacy. Furthermore, using multiple types of sensors has become the recent trend in FOG and fall detection in patients with PD. Nevertheless, the limitations in the current studies were obvious. The research was carried out with a low number of samples. A universally recognized adequate standard provoking FOG and fall is yet lacking, it might lead researchers to encounter difficulties in finding the best system based on the reported validation performance. Besides, there is little consensus on algorithm analysis. Future work should give careful consideration to address these limitations. First, an adequately studied population should be provided to support their study. Second, a consensus on provoking FOG/fall, methods of assessing validity and algorithm are necessary. Lastly, studies should carry out in a free-living environment with low-cost and low-energy consumption apparatus.

## Data availability statement

The original contributions presented in the study are included in the article/Supplementary material, further inquiries can be directed to the corresponding author.

## Author contributions

JH and TH conceived and designed the methodology of the systematic review. TH and ML extracted and collected the relevant information. TH drafted the manuscript. JH supervised the study at different steps and reviewed and edited the manuscript. All authors contributed to the article and approved the submitted version.

## Conflict of interest

The authors declare that the research was conducted in the absence of any commercial or financial relationships that could be construed as a potential conflict of interest.

## Publisher’s note

All claims expressed in this article are solely those of the authors and do not necessarily represent those of their affiliated organizations, or those of the publisher, the editors and the reviewers. Any product that may be evaluated in this article, or claim that may be made by its manufacturer, is not guaranteed or endorsed by the publisher.
